# Ferritin‐Based Nanocomposite Hydrogel Promotes Tumor Penetration and Enhances Cancer Chemoimmunotherapy

**DOI:** 10.1002/advs.202305217

**Published:** 2023-11-29

**Authors:** Rong Liu, Qian Liang, Jia‐Qi Luo, Yu‐Xuan Li, Xin Zhang, Kelong Fan, Jin‐Zhi Du

**Affiliations:** ^1^ School of Medicine South China University of Technology Guangzhou 510006 China; ^2^ CAS Engineering Laboratory for Nanozyme National Laboratory of Biomacromolecules Institute of Biophysics Chinese Academy of Sciences Beijing 100101 China; ^3^ School of Biomedical Sciences and Engineering Guangzhou International Campus South China University of Technology Guangzhou 511442 China; ^4^ University of Chinese Academy of Sciences Beijing 101408 China; ^5^ Nanozyme Medical Center School of Basic Medical Sciences Zhengzhou University Zhengzhou 450052 China; ^6^ National Engineering Research Center for Tissue Restoration and Reconstruction South China University of Technology Guangzhou 510006 China; ^7^ Key Laboratory of Biomedical Materials and Engineering Ministry of Education Guangdong Provincial Key Laboratory of Biomedical Engineering South China University of Technology Guangzhou 510006 China

**Keywords:** cancer chemoimmunotherapy, ferritin, hydrogel, transcytosis, tumor penetration

## Abstract

Hydrogels are prevailing drug delivery depots to improve antitumor efficacy and reduce systemic toxicity. However, the application of conventional free drug‐loaded hydrogel is hindered by poor drug penetration in solid tumors. Here, an injectable ferritin‐based nanocomposite hydrogel is constructed to facilitate tumor penetration and improve cancer chemoimmunotherapy. Specifically, doxorubicin‐loaded human ferritin (Dox@HFn) and oxidized dextran (Dex‐CHO) are used to construct the injectable hydrogel (Dox@HFn Gel) through the formation of pH‐sensitive Schiff‐base bonds. After peritumoral injection, the Dox@HFn Gel is retained locally for up to three weeks, and released intact Dox@HFn gradually, which can not only facilitate tumor penetration through active transcytosis but also induce immunogenic cell death (ICD) to tumor cells to generate an antitumor immune response. Combining with anti‐programmed death‐1 antibody (αPD‐1), Dox@HFn Gel induces remarkable regression of orthotopic 4T1 breast tumors, further elicits a strong systemic anti‐tumor immune response to effectively suppress tumor recurrence and lung metastasis of 4T1 tumors after surgical resection. Besides, the combination of Dox@HFn Gel^L^ with anti‐CD47 antibody (αCD47) inhibits postsurgical tumor recurrence of aggressive orthotopic glioblastoma tumor model and significantly extends mice survival. This work sheds light on the construction of local hydrogels to potentiate antitumor immune response for improved cancer therapy.

## Graphical abstract

In this study, a ferritin (HFn)‐based nanocomposite hydrogel is designed for cancer chemoimmunotherapy. The hydrogel can release intact drug‐loaded HFn nanocage in vivo and thus improve the penetration into tumor parenchyma through its active transcytosis and generates an immunostimulatory tumor microenvironment through ICD effect. The hydrogel shows superior antitumor efficacy in aggressive 4T1 breast and GL261 glioblastoma tumor models.

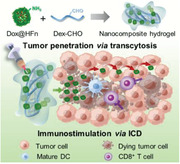

## 1 Introduction

Recently, cancer immunotherapy has made significant strides in clinical cancer treatment by stimulating the host's immune system to attack tumor cells and establishing long‐term immune memory.^[^ ^1^ ^]^ Particularly, immune checkpoint blockade (ICB) has considerably changed the therapeutic outcome of cancer patients.^[^ ^2^ ^]^ However, the response rate of immune checkpoint inhibitors such as anti‐programmed death‐1 antibody (αPD‐1) is usually <30%.^[^ ^3^ ^]^ One of the key reasons is that many tumors are immunologically cold tumors that develop highly immunosuppressive tumor microenvironments.^[^ ^4^ ^]^ To enhance the efficacy of ICB therapy, it is necessary to convert “cold” tumors into “hot” ones by enhancing the immunogenicity and remodeling the tumor immunosuppressive microenvironment. Immunogenic cell death (ICD) of tumor cells has been proven to be an important tactic to enhance tumor immunogenicity.^[^ ^5^, ^6^, ^7^ ^]^ A portion of classical chemotherapy agents, such as doxorubicin (Dox), not only kill cancer cells directly but also cause ICD effect to tumor cells, which can induce systemic immune response for cancer therapy.^[^ ^8^, ^9^ ^]^ However, systemic administration of these drugs typically results in severe side effects to normal organs. In addition, they can also bring harm to immune cells in bone marrows, and cause potential immunosuppression.^[^ ^10^ ^]^

Local administration of immunotherapeutic agents can initiate systemic immune responses against tumors, which could potentially generate greater efficacy and higher safety in comparison with systemic administration.^[^ ^11^, ^12^, ^13^ ^]^ Hydrogels have proven to be an ideal delivery system for a variety of immunotherapeutic drugs.^[^ ^14^, ^15^, ^16^, ^17^, ^18^, ^19^ ^]^ Injectable hydrogels can be used to concentrate drugs at the tumor site through minimally invasive methods.^[^ ^20^, ^21^ ^]^ They can achieve sustained drug release and maintain high local drug concentrations over a long period of time at the tumor site.^[^ ^22^ ^]^ For example, Liu et al. have developed an alginate hydrogel loaded with anticancer drugs and Toll‐like receptor 7 agonist imiquimod (R837) to trigger systemic immune response and effectively inhibit tumor metastasis and recurrence.^[^ ^23^ ^]^ Although traditional free drug‐loaded hydrogels have shown great promise for local delivery of various therapeutic agents, the tumor microenvironment presents obstacles hindering the effectiveness of this approach. Insufficient blood supply, high‐density extracellular matrix, and increased interstitial fluid pressure collectively limit the deep tumor penetration of drug molecules released from the hydrogel.^[^ ^24^, ^25^, ^26^ ^]^ It is crucial to develop novel penetrative hydrogels to improve drug penetration in solid tumors. To this end, Cui et al. developed a series of supramolecular hydrogels consisting of tissue‐penetrating cyclopeptides (eg. iRGD) and therapeutic drugs to enhance tumor penetration and improve the efficacy of chemoimmunotherapy in a variety of tumor models.^[^ ^27^, ^28^, ^29^, ^30^ ^]^ These studies highlight the importance and potency of such hydrogels for local cancer chemoimmunotherapy. Despite these advances, it should be noted that the tumor‐penetrative performance of previous systems was mainly achieved by the aid of well‐known penetrative molecules such as iRGD. Developing injectable hydrogels using materials with intrinsic tumor‐penetrative property can be a promising alternative strategy, which will also simplify the recipe and preparation of the hydrogels.

Transcytosis is a nature transcellular transport process in which macromolecules are transported from one side of a cell to the other.^[^ ^31^ ^]^ Recently, transcytosis has received increasing attention for nanoparticle‐based drug delivery. It has been suggested that up to 97% of the entry of nanoparticles into solid tumors depends on the active transcytosis process through endothelial cells.^[^ ^32^ ^]^ In addition, sophisticated nanomedicines have shown effective penetration into solid tumors by means of active transcytosis, which generated extraordinary antitumor efficacy.^[^ ^32^, ^33^, ^34^, ^35^, ^36^, ^37^, ^38^ ^]^ However, the underlying mechanism to develop well‐defined nanoparticles with active transcytosis capability has been unclear. Ferritin, a natural protein with an outer diameter of 12 nm and an inner diameter of 8 nm, has been widely studied by us and others as drug delivery carriers due to its cage‐like structure and intrinsic affinity to the transferrin receptor 1 (TfR‐1) on many tumor cells.^[^ ^39^, ^40^, ^41^, ^42^ ^]^ Our recent studies have for the first time revealed that ferritin was able to pass through the blood–brain barrier via transcytosis in brain endothelial cells.^[^ ^43^ ^]^ However, the transcytosis transport of ferritin through tumor cells as well as the inclusion of ferritin to construct injectable penetrative hydrogels for cancer immunotherapy has not been reported.

**Figure 1:**
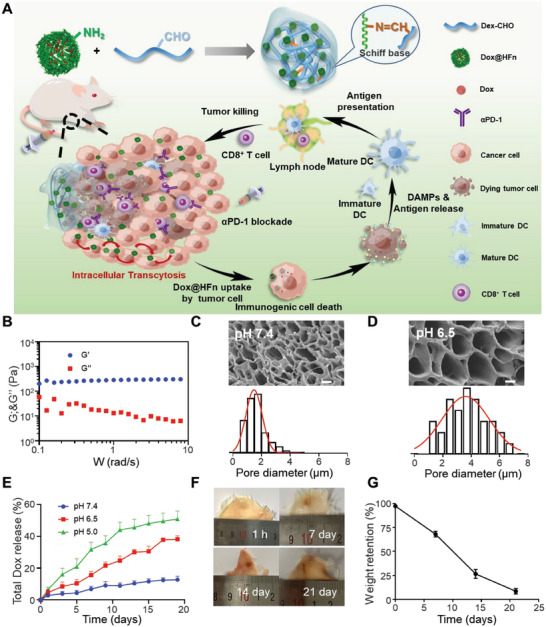
Preparation and characterization of Dox@HFn Gel. A) Schematic illustration of the formulation and working mechanism of Dox@HFn Gel. B) The storage modulus (G’) and loss (G’’) modulus of HFn Gel^L^. C,D) SEM images and diameter analysis of HFn Gel^L^ after swelling in PBS at pH 7.4 (C) and pH 6.5 (D). Scale bars: 2 µm. E) In vitro cumulative release of total Dox from Dox@HFn Gel^L^ at pH 7.4, 6.5, and 5.0, respectively (*n* = 3). F) Representative photographs of HFn Gel^L^ at different time points after subcutaneous injection in mice. G) Quantitative analysis of remaining weights of HFn Gel^L^ at 1 h, and on 7 days, 14 days, and 21 days (*n* = 3). Statistical data are presented as means ± SD.

In the present study, we constructed an injectable nanocomposite hydrogel consisting of Dox‐loaded ferritin and oxidized dextran (Dex‐CHO) for improved cancer chemo‐immunotherapy (**Figure** **1**A). The hydrogel is formed through chemical crosslinking of Dex‐CHO and Dox‐loaded human heavy chain ferritin (Dox@HFn). The gel precursors are mixed and locally injected into the peritumoral site where they form Dox@HFn Gel in situ to enhance Dox retention at the tumor site. The hydrogel can be degraded gradually due to the presence of Schiff base bonds and releases intact Dox@HFn in the acidic tumor microenvironment. Our study revealed that Dox@HFn shows transcytosis in tumor cells and can thus deeply penetrate into the tumor parenchyma through active transcytosis. Dox@HFn can be internalized by tumor cells to induce the ICD effect and generate an immunostimulatory tumor microenvironment in the notorious 4T1 triple‐negative breast tumor model. When the Dox@HFn Gel was combined with an immune checkpoint inhibitor such as αPD‐1, this chemoimmunotherapy effectively inhibited tumor growth, recurrence, and metastasis of 4T1 breast cancer. More interestingly, the combination of Dox@HFn Gel with anti‐CD47 antibody (αCD47) significantly extended mice survival in the orthotropic glioblastoma tumor model. By constructing ferritin‐based nanocomposite hydrogel, our findings shed light on the potential of localized hydrogel constructs in enhancing the antitumor immune response for improved cancer immunotherapy.

## 2 Results and Discussion

### 2.1 Synthesis and Characterization of Dox@HFn Gel

In our study, the injectable hydrogel was prepared by the reaction of aldehyde‐modified dextran (Dex‐CHO) with amino groups of HFn. Dex‐CHO was prepared by oxidizing dextran with sodium periodate (Figure S1A, Supporting Information). Colorimetric analysis indicated that the degree of oxidation was ≈33% (Figure S1B,C, Supporting Information). HFn and doxorubicin‐loaded HFn (Dox@HFn) were synthesized as previously described.^[^ ^43^ ^]^ The size of HFn and Dox@HFn was ≈12 nm with narrow size distributions according to dynamic light scattering (DLS) measurement (Figure S2A,B, Supporting Information). The loading content of Dox in Dox@HFn was determined to be 9.21% (w/w) according to the UV–vis absorbance measurement. The blank hydrogel (HFn Gel) without Dox loading was prepared by mixing Dex‐CHO with HFn. The gelation time of HFn Gel could be adjusted by changing the concentration of Dex‐CHO. We fixed the concentration of HFn at 200 mg mL^−1^ and selected Dex‐CHO solutions at high (120 mg mL^−1^) and low (90 mg mL^−1^) concentrations to prepare HFn Gel^H^ (gelation time ca. 5 min) and HFn Gel^L^ (gelation time ca. 11 min), respectively (Table S1, Supporting Information).

Next, the viscoelasticity of HFn Gel^H^ and HFn Gel^L^ was measured. As shown in Figure 1B and Figure S3A (Supporting Information), the storage modulus (G′) of each hydrogel was independent of the frequency over the range of 0.1–10 Hz, while the loss modulus (G″) slightly decreased with the frequency increased. Moreover, it is observed that G’ was greater than G’’ across the swept frequency, indicating that HFn Gel exhibits characteristic behavior and elastic dominant properties of solids. The G’ and G’’ values of HFn Gel^H^ were much higher than that of HFn Gel^L^, which was reasonable since HFn Gel^H^ had higher crosslinking density. The thixotropic behavior indicated that HFn Gel^H^ collapsed at a strain rate of 20% (Figure S3B, Supporting Information), while HFn Gel^L^ collapsed at a strain rate of 110% (Figure S3C, Supporting Information). The swelling curve showed that HFn Gel^L^ reached equilibrium swelling ratio ≈256% ± 17% in PBS pH 7.4 (Figure S3D, Supporting Information). Due to the formation of Schiff base bonds between Dex‐CHO and HFn, the hydrogel may show pH‐dependent swelling behaviors. To test this, HFn Gel^L^ was incubated in PBS at pH 7.4 or 6.5 for 24 h. After lyophilization, their morphologies were observed by scanning electron microscope (SEM). As indicated in Figure 1C,D, the pore size of the hydrogel was ≈1.6 µm after swelling at pH 7.4, while the pore size expanded to 3.7 µm at pH 6.5. This might be due to the partial cleavage of Schiff base bonds of the hydrogel network in acidic environments.

Dox‐loaded hydrogel (Dox@HFn Gel) was prepared by mixing Dex‐CHO and Dox@HFn at the doses as the same as the preparation of HFn Gel^L^. Dox@HFn Gel^L^ were placed in buffers of pH 7.4, 6.5, or 5.0 to simulate normal condition, mild acidic tumor microenvironment, and acidic endosomal compartment, respectively. The results showed that the total release rate of Dox increased significantly with pH decrease. The cumulative release of Dox is 12.73% ± 1.77%, 38.12 ± 1.80%, and 50.83% ± 4.99% on the 19th day at pH 7.4, 6.5 and 5.0, respectively, indicating that the hydrogel can work as a drug depot for sustained Dox release (Figure 1E). One key design of Dox@HFn Gel is that it can release intact Dox@HFn nanocages for improved tumor penetration. To prove Dox@HFn Gel^L^ could release intact Dox@HFn at pH 6.5, the release solution was subjected to ultrafiltration using an ultrafiltration tube with molecular weight cutoff of 10 kDa, which allowed the filtration of free Dox but not Dox@HFn. As can be seen, the upper layer solution was red and the lower layer solution was almost colorless (Figure S4A, Supporting Information). UV–vis measurement indicated that the content of Dox in the upper solution was significantly higher than that in the lower solution (Figure S4B, Supporting Information), indicating that most of the released Dox was in the form of Dox@HFn rather than free Dox. After measuring the Dox absorbance in the upper and lower layer solutions by an UV–vis spectrophotometer, we calculated the cumulative release of free Dox was 12.22 ± 0.45%, while the release of Dox@HFn was 25.90 ± 2.16% (Figure S4C, Supporting Information). DLS also indicated the presence of HFn in the upper layer solution (Figure S4D, Supporting Information).

Furthermore, we injected a mixed solution of Dex‐CHO and HFn subcutaneously into the back of BALB/c mice to evaluate the degradation of HFn Gel^L^ in vivo. It was observed that the mixed solution formed hydrogel in situ in the subcutaneous area (Figure 1F), and became smaller and smaller with time extension. The remaining hydrogel was peeled off and weighted at designated time points to quantitatively calculate its degradation kinetics. As shown in Figure 1G, HFn Gel^L^ showed ca. 32% weight loss after 1 week and lose ca. 92% of its original weight on 21 days. In this experiment, we could see that the hydrogel did not bring harms to the mouse at the injection site in spite of high local concentrations. Moreover, the hemolysis testing also indicated that HFn Gel^L^ did not show detectable hemolysis (Figure S5A,B, Supporting Information).

### 2.2 In Vitro Cellular Uptake, Transcytosis, and Penetration of Dox@HFn

**Figure 2:**
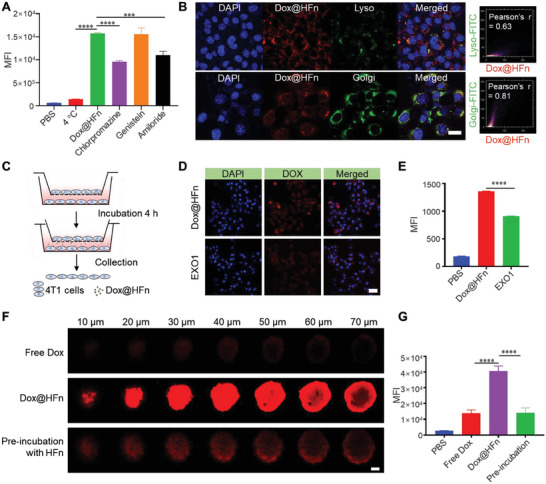
In vitro cellular uptake, transcytosis, and penetration of Dox@HFn. A) Cellular uptake of Dox@HFn by 4T1 cells after pretreatment with various endocytosis inhibitors for 1 h (*n* = 3). B) Colocalization of Dox@HFn with lysosome (green) and Golgi apparatus (green) at 4 h incubation. Scale bars: 20 µm. ImageJ was utilized to perform Pearson's correlation coefficient analysis to determine the co‐localization of green and red. C) Schematic illustration of transcytosis of Dox@HFn in vitro. D) CLSM images and E) flow cytometry analysis of 4T1 cells on the lower chamber (*n* = 3). Scale bars: 50 µm. F) CLSM images and G) flow cytometry analysis of the 4T1 cell‐derived multicellular spheroids (MSCs) with various treatments for 4 h (*n* = 3). Scale bars: 50 µm. Statistical data are presented as means ± SD. ^***^ *P* < 0.001, ^****^ *P* < 0.0001.

Ferritin nanocage, in comparison to synthetic nanomedicine, can actively bind to specific receptors on tumor cells to enhance their cellular uptake.^[^ ^39^ ^]^ Therefore, this unique property makes ferritin nanocage a promising candidate as targeted drug delivery carrier for cancer treatment. We verified that the expression of Tim‐2 receptors on 4T1 cells using flow cytometry and Western blot (Figure S6, Supporting Information). Then, the cellular uptake of Dox and Dox@HFn by 4T1 cells was analyzed by flow cytometry and confocal laser scanning microscope (CLSM). As shown in Figure S7A (Supporting Information), increasing Dox and Dox@HFn were taken up by the cells with incubation time extension. CLSM images indicated that free Dox primarily entered cell nucleus, while Dox@HFn was mainly distributed in the cytoplasm (Figure S7B, Supporting Information). We then investigated the endocytosis pathway of Dox@HFn using specific endocytosis inhibitors. As shown in **Figure** **2**A, the cellular uptake of Dox@HFn decreased by 38% and 30% using chlorpromazine and amiloride, respectively. This suggests that the cellular uptake of Dox@HFn by 4T1 cells may rely on clathrin‐ and macropinocytosis‐mediated endocytosis. To further understand the internalization of Dox@HFn, we used CLSM to observe the distribution of Dox@HFn in lysosome and Golgi apparatus in 4T1 cells (Figure 2B). The results showed that Dox@HFn co‐localized well with the Golgi apparatus with the Pearson correlation coefficient as high as 0.81. This indicates that Dox@HFn can be sorted to the Golgi apparatus after internalization, which is believed to facilitate rapid extracellular efflux of the cargoes by exocytosis.^[^ ^44^ ^]^

To verify if transcytosis occurs for Dox@HFn in 4T1 tumor cells, the transwell culture system was adopted (Figure 2C). 4T1 cells were pretreated with Dox@HFn alone or with an exocytosis inhibitor EXO1 for 4 h in 24‐well plates. Then, the cells were collected and seeded in the upper chamber of the transwell system, while blank 4T1 cells were seeded in the lower chamber. After incubation for additional 4 h, the fluorescence of Dox@HFn in 4T1 cells in the lower chamber was analyzed by CLSM and flow cytometry. As indicated in the CLSM images, dense red fluorescence from Dox@HFn could be observed in the lower layer 4T1 cells under normal incubation condition, indicating that Dox@HFn can transported from the upper chamber to the low chamber by transcytosis. When the upper layer 4T1 cells were pretreated with an exocytosis inhibitor EXO1, the red fluorescence in the lower layer 4T1 cells significantly weakened, probably because the exocytosis of Dox@HFn was inhibited (Figure 2D). Quantitative analysis by flow cytometry was consistent with CLSM imaging that EXO1 pretreatment reduced the mean fluorescence intensity (MFI) of 4T1 cells in the lower chamber (Figure 2E). These results collectively suggest that HFn can realize transcellular transport by active transcytosis in 4T1 tumor cells.

We presume that such an active transcytosis mechanism may facilitate deep penetration of Dox@HFn in tumors. To test this, the penetration capability of Dox and Dox@HFn was first studied in 4T1 cell‐derived multicellular spheroids (MCSs). Free Dox and Dox@HFn at an equivalent dose of Dox were incubated with MCSs for 4 h. Confocal images indicated that free Dox was mainly distributed at the periphery of the MCSs, while Dox@HFn could penetrate deeply into the spheroid according to the Z‐axis scanning (Figure 2F). After digesting the MCSs into single cells and subjecting to flow cytometry analysis, we found that the MFI of Dox@HFn treated cells was much higher than that after Dox treatment (Figure 2G). Pretreatment of the MCSs with excess blank HFn significantly reduced the penetration of Dox@HFn (Figure 2F,G). The improved penetration capability of Dox@HFn than free Dox may be achieved via Tim‐2‐dependent transcytosis in tumor cells. The MCSs were pretreated with either the endocytosis or the exocytosis inhibitors, and the penetration depth of Dox@HFn was reduced (Figure S8A, Supporting Information). Flow cytometry analysis also showed that the MFI of inhibitor‐treated cells was significantly lower than the untreated cells (Figure S8B, Supporting Information). These results demonstrate that the intrinsic transcytosis property of HFn facilitates a deep penetration in MCSs, which makes it attractive for the fabrication of penetrative hydrogels for local cancer treatment.

### 2.3 In Vitro Immunogenic Cell Death Effect of Dox@HFn

We then investigated the cell‐killing effect of Dox@HFn against 4T1 cells via the MTT and apoptosis assays. As shown in Figure S9 (Supporting Information), both free Dox and Dox@HFn could kill 4T1 tumor cells after 48 h treatment. The apoptosis rates for 4T1 cells after treatments of Dox at 2 and 5 µm or Dox@HFn at an equivalent Dox concentration of 5, 10, and 15 µm were 84.33%, 83.6%, 49.53%, 66.93%, and 68.33%, respectively (Figure S10, Supporting Information). It should be noted that free Dox is more effective in killing 4T1 cells in both the MTT and apoptosis assays, which may be due to that free Dox can enter cell nuclei directly, whereas Dox@HFn enters cell cytoplasm first and takes time to release free Dox to kill cells.

**Figure 3:**
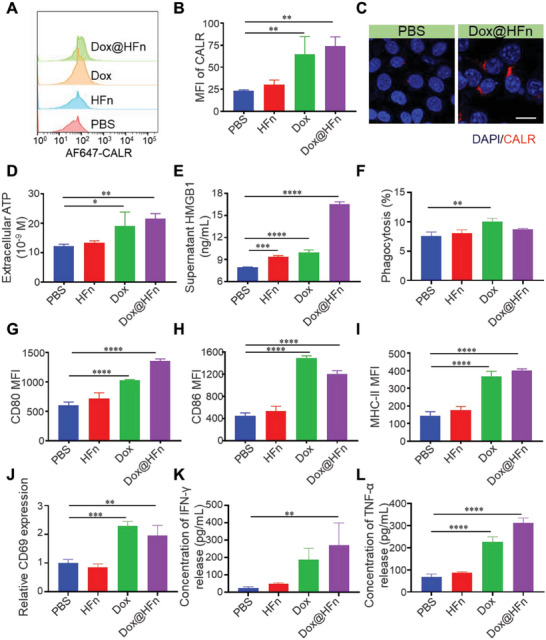
Dox@HFn induced ICD of 4T1 tumor cells in vitro. A) Flow cytometric examination of CALR expression on 4T1 cell surface after incubation with PBS, HFn, Dox, and Dox@HFn for 24 h. B) MFI of 4T1 cells after the treatments (*n* = 3). C) CLSM images of CALR expression (red) on 4T1 cells after various treatments for 24 h. Scale bars: 20 µm. D) ATP and E) HMGB1 secretion in culture media of 4T1 cells after various treatments for 24 h (*n* = 3). F) The phagocytosis of BMDCs to 4T1 cells after various treatments (*n* = 3). BMDCs were co‐cultured with 4T1 cells for 24 h. G–I) The CD80, CD86, and MHC‐II expressions of BMDCs co‐cultured with 4T1 cells after various treatments (*n* = 3). J) Relative CD69 expression on CD8^+^ T cells treated with BMDCs for 24 h after different treatments. K) IFN‐γ and L) TNF‐α release from T cells induced by BMDCs after different treatments. Statistical data are presented as means ± SD. *P < 0.05, **P < 0.01, ***P < 0.001, ****P < 0.0001.

It has been found that traditional chemotherapy agents such as Dox can induce ICD to tumor cells.^[^ ^45^ ^]^ Then, the dying tumor cells release damage‐associated molecule patterns (DAMPs), including calreticulin (CALR), high mobility group protein B1 (HMGB1) and adenosine triphosphate (ATP).^[^ ^46^ ^]^ DAMPs can serve as adjuvants to augment the specific immune response against tumors.^[^ ^47^ ^]^ They can activate the maturation of dendritic cells as well as facilitate antigen presentation, leading to the initiation of T cell proliferation and differentiation. We then studied the ability of Dox@HFn to induce the ICD effect in 4T1 cells. 4T1 cells were treated with 2 µm Dox and 10 µm Dox@HFn for 24 h, respectively. The expression of CALR on the 4T1 cell membrane was quantified and visualized by flow cytometry and CLSM. The results of flow cytometry showed that blank HFn had almost no effect on the expression of CALR, while Dox and Dox@HFn significantly increased the cell surface exposure of CALR. The expression level of CALR was increased by 2.8 times and 3.2 times, respectively, compared to the untreated group (**Figure** **3**A,B). CLSM images confirmed the exposure of CALR on cell surface after Dox@HFn treatment (Figure 3C). After Dox@HFn treatment, the HMGB1 was transferred from the nucleus to the cytoplasmic or extracellular environment and ATP secretion in the culture medium of 4T1 cells increased (Figure 3D,E).

We further investigated whether Dox@HFn‐treated 4T1 cells could promote the phagocytosis and activation of bone marrow‐derived dendritic cells (BMDCs). 4T1 cells pretreated with Dox or Dox@HFn were co‐cultured with BMDCs, and their phagocytosis of 4T1 cells was detected by flow cytometry. It was found that the basal phagocytosis of BMDCs toward 4T1 cells is 7.56%, while the phagocytosis of 4T1 cells pretreated with free Dox and Dox@HFn increased to 10.05% and 8.74%, respectively (Figure 3F). Besides, Dox@HFn treatment also increased the phagocytosis of 4T1 cells by bone marrow‐derived macrophages (BMDMs) (Figure S11, Supporting Information). Free Dox and Dox@HFn significantly increased the expression of CD80, CD86, and MHC‐II on the surface of BMDCs (Figure 3G–I), indicating the activation and maturation of BMDCs. Multiple dendritic‐like protrusions on BMDCs can be observed under optical microscope after they were co‐incubated with Dox@HFn‐treated 4T1 cells (right panel) (Figure S12, Supporting Information).

To determine whether activated BMDCs promote T cell activation, the expression of activation markers (such as CD69) on CD8^+^ T cells and the release of cytokines (interferon‐gamma (IFN‐γ) and tumor necrosis factor‐alpha (TNF‐α)) from T cells were studied after co‐culturing T cells and BMDCs at 10:1 for 24 h. As indicated in Figure 3J, Dox@HFn treatment induced a significantly higher expression of CD69 among CD8^+^ T cells. Meanwhile, the levels of IFN‐γ and TNF‐α secreted by T cells were significantly elevated after Dox@HFn treatment (Figure 3K,L).

### 2.4 Tumor Penetration and In Vivo Antitumor Activity of Dox@HFn against 4T1 Tumors

**Figure 4:**
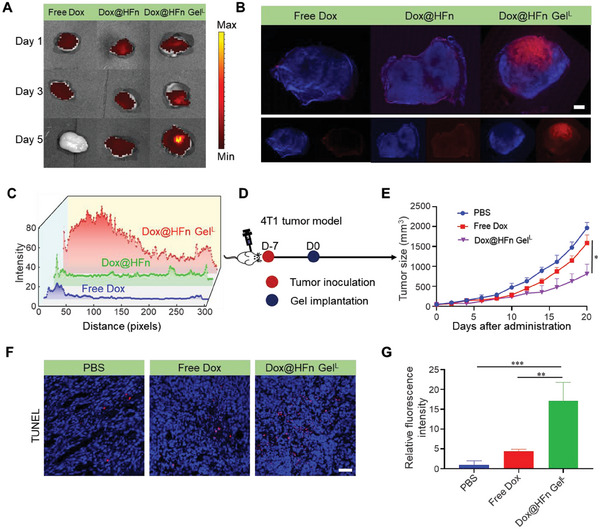
Dox@HFn Gel improves tumor penetration and therapeutic efficacy in orthotopic 4T1 tumors. A) Dox fluorescence signal in tumor tissue after peritumoral injections of free Dox, Dox@HFn, and Dox@HFn Gel^L^, respectively. B) CLSM imaging of tumor tissue sections on day 5 after peritumoral injections. Red fluorescence represents Dox; blue fluorescence represents cell nuclei stained with 4′,6‐diamidino‐2‐phenylindole (DAPI). Scale bars: 1 mm. C) Semi‐quantitative analysis of red fluorescence intensity of tumor tissues after various treatments as a function of the distance from the injection site. D) Schematic illustration of the treatment schedule. E) Average tumor growth curves of 4T1 tumor‐bearing mice after various treatments (*n* = 5). F) TUNEL staining of tumor tissue sections after various treatments. Scale bars: 50 µm. G) Relative fluorescence intensity of TUNEL‐positive cells was measured per field by Image J (*n* = 3). Statistical data are presented as means ± SD. *P < 0.05, **P < 0.01, ***P < 0.001.

Considering the promising properties of Dox@HFn in tumor penetration and ICD effect, we would like to evaluate the in vivo performance and antitumor activity of the nanocomposite hydrogel Dox@HFn Gel^L^ in 4T1 tumors. We first compared the retention and penetration capability of Dox@HFn Gel^L^ with free Dox and Dox@HFn in tumor tissues after peritumoral injection. On day 5 after injection, no fluorescent signal was detected in the group of receiving free Dox (**Figure** **4**A). The fluorescence signal of Dox@HFn gradually weakened from day 1 to day 5. However, the fluorescence signal of Dox@HFn Gel^L^ gradually increased over time. These results suggest that free Dox and Dox@HFn may leak out easily from the tumor site, whereas Dox@HFn Gel^L^ manages to enhance the local retention and accumulation of Dox in the tumor tissue (Figure 4A).

To further test our hypothesis that Dox@HFn Gel^L^ could improve the penetration of Dox deeply into the tumor tissue, we collected the treated tumors on the 5th day and performed CLSM examination. In mice treated with free Dox, only minimal fluorescence was detected throughout the tumor. In contrast, Dox@HFn Gel^L^ and Dox@HFn treated tumors exhibited a much more significant fluorescence signal throughout the tumor tissue (Figure 4B). We measured the fluorescence intensity of Dox to quantitatively compare their tumor penetration, and plotted the fluorescence intensity of Dox as a function of the distance from the injection site. Compared with tumors treated with free Dox and Dox@HFn, Dox@HFn Gel^L^ exhibited stronger fluorescence and showed a greater Dox diffusion distance (Figure 4C). These results suggest that Dox@HFn Gel^L^ can facilitate drug retention and penetration deeper into tumor tissue. We also found that HFn managed to reach and accumulate in hypoxic tumor areas lacking blood vessels after intravenous administration (Figure S13, Supporting Information). Encouraged by these results, we preliminarily evaluated the anticancer efficacy of the Dox@HFn Gel^L^ in orthotopic 4T1 tumor model (Figure 4D). As shown in Figure 4E, tumor growth was inhibited in mice treated with free Dox, but mice treated with Dox@HFn Gel^L^ showed more significant suppression of tumor growth. This is reasonable since the hydrogel prolongs drug retention and improves tumor penetration. Terminal deoxynucleotide transferase dUTP nick‐end labeling (TUNEL) assay indicated that Dox@HFn Gel^L^ induced most apoptotic cells (Figure 4F,G).

### 2.5 Combination Therapy of Dox@HFn with αPD‐1 against Orthotopic 4T1 Tumors

**Figure 5:**
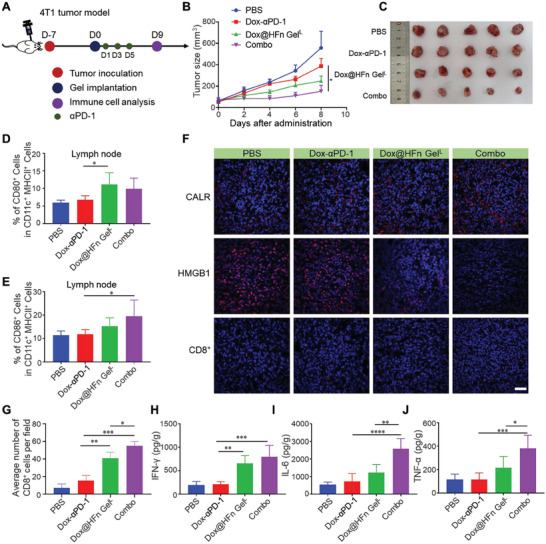
Combination therapy of Dox@HFn Gel^L^ with αPD‐1 in orthotopic 4T1 tumor model. A) Schematic illustration of the combination therapy. B) Average tumor growth curves of 4T1 tumor‐bearing mice after various treatments (*n* = 5). The treatments include: PBS, Dox‐αPD‐1: free Dox plus αPD‐1, Dox@HFn Gel^L^, and Combo: Dox@HFn Gel^L^ plus αPD‐1. C) Representative tumor photographs from mice on the 9th day after treatment. D,E) Percentages of CD80^+^ (D) and CD86^+^ cells (E) gated from CD11c^+^ MHC‐II^+^ cells in lymph node (*n* = 5). F) CLSM images showing the expression of CALR and HMGB1 as well as the infiltration of CD8^+^ T cells in tumor tissues. Scale bars: 50 µm. G) Quantitative analysis of numbers of CD8^+^ T cells infiltrated in tumors. Enzyme‐linked immunosorbent assay (ELISA) detection of H) IFN‐γ, I) IL‐6, and J) TNF‐α levels in tumor tissues on the 9th day after treatment (*n* = 5). Statistical data are presented as means ± SD. *P < 0.05, **P < 0.01, ***P < 0.001.

It is known that ICD effect can sensitize the tumors to immune checkpoint inhibitor treatment.^[^ ^48^ ^]^ To test this, we studied the combination therapy of Dox@HFn with anti‐programmed death‐1 antibody (αPD‐1) against orthotopic 4T1 tumors (**Figure** **5**A). First, 4T1 tumor cells were inoculated into the mammary fat pad of female BALB/c mice. When the tumor volume reached 50–100 mm^3^, the tumor‐bearing mice were randomly divided into four groups and treated with PBS, free Dox plus αPD‐1 (Dox‐αPD‐1), Dox@HFn Gel^L^, and Dox@HFn Gel^L^ plus αPD‐1 (Combo). The mice received three consecutive intraperitoneal injections of αPD‐1 after Dox@HFn Gel^L^ implantation. The tumor growth inhibition curve of each group is presented in Figure 5B. As indicated, the tumor inhibitory rate of Dox‐αPD‐1 and Dox@HFn Gel^L^ was 30.6% and 55.3%, respectively. In contrast, the Combo treatment significantly delayed tumor growth compared to other treatments with the tumor inhibitory rate of 72.7%. The average body weight of the mice in each group remained relatively stable throughout the study (Figure S14A, Supporting Information). Furthermore, the tumor weight at the end of the treatment confirmed its therapeutic effect (Figure 5C; Figure S14B, Supporting Information).

It is known that dendritic cells (DCs) are the important antigen‐presenting cells (APCs), which play a major role in regulating innate and adaptive immunity.^[^ ^6^ ^]^ To evaluate the immunostimulatory effect of the hydrogel, we assessed the expression levels of surface activation markers on DCs in lymph nodes on the 9th day after treatment. As shown in Figure 5D,E, treatment with Dox@HFn Gel^L^, with or without αPD‐1, significantly increased the percentage of CD86^+^ and CD80^+^ cells among CD11c^+^MHCII^+^ DCs. Tumors were collected on the 9th day after various treatments and analyzed by immunofluorescence staining. The expression of CALR was increased in tumors treated with Combo compared to other treatments (Figure 5F). Mice treated with Combo showed a decrease in HMGB1 secretion and significant infiltration of CD8^+^ T cells (Figure 5F,G). Inflammatory cells are known to produce interferon‐gamma (IFN‐γ), interleukin (IL)−6, and tumor necrosis factor‐alpha (TNF‐α), all of which play vital roles in the immune response. Enzyme‐linked immunosorbent assay (ELISA) measurement showed that Combo treatment significantly elevated the secretion of IFN‐γ, IL‐6, and TNF‐α by 4.05, 4.74, 3.34 times in comparison with PBS treatment (Figure 5H–J). These results suggest that combination therapy can remodel the tumor immunosuppressive microenvironment and enhance the effectiveness of tumor treatment.

### 2.6 Long‐Term T Cell Memory Response and Prevention of Postsurgical Tumor Recurrence of Combination Therapy

**Figure 6:**
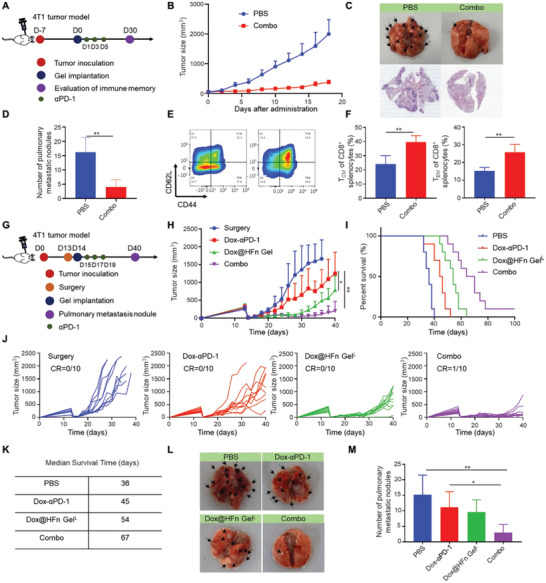
Long‐term immune response of combination therapy in orthotopic 4T1 tumor model. A) Schematic illustration of memory study. B) Tumor growth curve for combination therapy in 4T1 breast tumor model (*n* = 6). C) Representative photographs and HE staining of the lungs at the end of treatment. D) Quantitative analysis of metastatic nodules on the lungs (*n* = 5). E) Representative flow cytometry chart and F) percentage of central memory T cells (T_CM_) and effective memory T cells (T_EM_) in splenocytes after treatment (*n* = 4). G) Treatment schedule in 4T1 postsurgical tumor model. H) Tumor growth curves of the mice after surgical resection and various treatments (*n* = 10). I) Percent survival of mice after various treatments (*n* = 10). J) Individual tumor growth kineties after various treatments (*n* = 10). K) Summary of survival time of mice after various treatments. L) Representative photographs and M) quantitative analysis of metastatic nodules on the lungs (*n* = 10). Statistical data are presented as means ± SD. *P < 0.05, **P < 0.01.

Tumor metastasis and recurrence account for major cancer deaths. It is essential to prevent tumor metastasis and recurrence by establishing long‐term immune memory. Thus, the long‐term T cell memory response was studied in the orthotopic 4T1 tumor model (**Figure** **6**A). Consistent with previous results, combination therapy effectively inhibited tumor growth (Figure 6B). Then, the lungs were collected on day 30 after the treatment and the number of pulmonary metastatic nodules in the lungs were quantified. As indicated, the lungs after Combo treatment showed significantly less metastatic nodules than that in the control group, indicating that Combo therapy effectively inhibited lung metastasis (Figure 6C,D). The spleen was also collected to evaluate whether Combo treatment could induce T cell memory response. Flow cytometry analysis indicated that the ratio of central memory T cells (T_CM_) and effector memory T cells (T_EM_) in the spleen has been increased to 39.5% and 25.8% after Combo treatment, which was significantly higher than 23.97% and 15.3% in the control (Figure 6E,F). These results indicate that a strong T cell memory has been formed after the combination therapy.

In terms of this, we hypothesize that the Combo treatment may prevent tumor recurrence and metastasis after surgery by inducing antitumor immune responses. We then constructed an incomplete surgical resection of the orthotopic 4T1 breast cancer model (Figure 6G). When the tumor volume reached 200–300 mm^3^, ≈90% of the tumor was surgically resected. The operated mice were randomly divided into four groups and given different treatments. As shown in Figure 6H–K, mice in the control group showed a rapid tumor recurrence with a median survival time of only 36 days after surgery. In contrast, the combination treatment effectively extended the survival time of mice compared to other treatments, and the median survival time after surgery was 67 days. It was worth noting that one of the mice in the combination treatment was completely cured of the tumor without recurrence (Figure 6J). Moreover, we collected the lung tissues of mice on the 40th day of tumor cell inoculation. It was evident that the other group had more metastatic nodules than the combination treatment (Figure 6L,M; Figure S15, Supporting Information). These results suggest that combination therapy inhibits the growth and lung metastasis of 4T1 breast cancer and produces a durable anti‐tumor immune memory.

### 2.7 Combination Therapy Suppressed Malignant Glioblastoma Recurrence Post‐Resection

**Figure 7:**
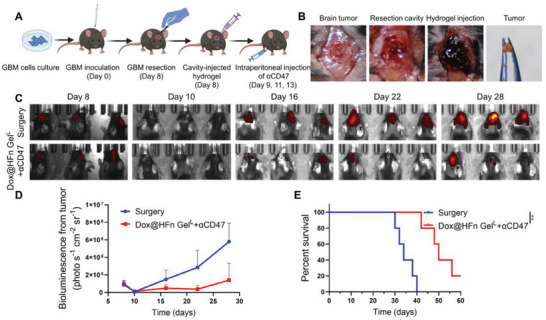
In vivo treatment of Dox@HFn Gel^L^ plus αCD47 in GL261 surgical glioblastoma tumor model. A) Schematic representation of the experimental design. Created with BioRender.com. B) Surgical tumor debulking and hydrogel cavity implantation on Day 8. C) Bioluminescence images of tumor recurrence after the treatment. D) Semi‐quantification of the bioluminescence signal intensity (*n* = 3). E) Percent survival of mice after the treatments (*n* = 5). Statistical data are presented as means ± SD. **P < 0.01.

Glioblastoma (GBM) is a highly invasive and malignant tumor that occurs in the central nervous system. Due to the infiltrative growth of GBM cells, it is challenging to eradicate tumor cells through surgical resection, and the residual GBM cells cause rapid recurrence.^[^ ^49^ ^]^ Given that tumor‐associated microglia/macrophages (TAMs) contribute 30–50% of the tumor mass in brain tumors, immunotherapy targeting TAMs holds great potential for improved GBM treatment.^[^ ^50^ ^]^ Encouraged by the positive results in surgical 4T1 tumor model, a GBM resection model was established to investigate the treatment potential of the combination of Dox@HFn Gel^L^ with anti‐CD47 antibody (αCD47). As shown in **Figure** **7**A, GL261‐Luc cells were inoculated on Day 0, and tumor resection was performed on Day 8. Then, the Dox@HFn Gel^L^ was injected immediately into the surgical cavity and αCD47 was intraperitoneally injected in the meantime (Figure 7B). The tumor recurrence was monitored through the bioluminescence imaging. Compared to the control surgery group, the bioluminescence signals of the mice receiving the combination treatment (Dox@HFn Gel^L^+αCD47) were much weaker until Day 28 (Figure 7C,D). The combination treatment also significantly extended the median survival of the mice from 34 days to 50 days (Figure 7E). These preliminary results indicate that the combination of the hydrogel formulation with αCD47 treatment holds great potential to reduce postoperative recurrence of malignant GBM.

## 3 Conclusion

In this study, we developed a ferritin‐based nanocomposite Dox@HFn Gel^L^ as a local drug‐releasing depot to improve cancer chemo‐immunotherapy via enhancing drug retention and penetration in solid tumors. After peritumoral injection, Dox@HFn Gel^L^ could maintain in the tumor site for several weeks, and gradually released Dox@HFn nanoparticles. This feature on one hand could prolong the retention time of Dox in the tumor site, which is beneficial to maintaining effective drug dose for longer time. On the other hand, the released Dox@HFn enabled active transcytosis in tumor cells. Such an active transcellular transport mechanism significantly facilitated Dox penetration into deep tumor parenchyma and thus elicited more prominent immunostimulatory response through ICD effect. Combination of Dox@HFn Gel^L^ with αPD‐1 significantly improved tumor‐infiltrating CD8^+^ T cells as well as elicited long‐term T cell memory immune response, which effectively inhibited postsurgical tumor recurrence and metastasis in orthotopic 4T1 tumor model. In addition, the combination of Dox@HFn Gel^L^ with αCD47 inhibited postsurgical tumor recurrence of the more aggressive glioblastoma tumor model and significantly extended the mice survival. Our results demonstrate that this local drug delivery system based on nanocomposite hydrogel holds great potential to improve cancer chemoimmunotherapy.

## 4 Experimental Section

### Materials

Dextran and sodium periodate were obtained from Aladdin Reagent Co., LTD (Shanghai, China). BCA assay kits and ATP assay kits were obtained from Beyotime Biotechnology (China). HMGB1 detection kits were purchased from Arigo Biolaborators (Shanghai, China). Biolegend BD bioscience flow cytometry antibodies were adopted. Anti‐Calreticulin‐AF647 was bought from Abcam. Hunan SJA Laboratory Animal Co., Ltd. (Changsha, China) supplied the female BALB/c mice. Mice were maintained in an SPF condition. All animal experiments were carried out following a protocol (2018018) approved by the Institutional Animal Care and Use Committee at South China University of Technology and complied with all relevant ethical regulations. The 4T1 cell line was a gift from Professor Jun Wang's lab at South China University of Technology, and was cultured in RPMI‐DMEM medium.

### Release of Dox from Dox@HFn Hydrogels

The hydrogel was formed through gelation by mixing the Dox@HFn solution with Dex‐CHO solution. To investigate the release behavior of Dox@HFn hydrogels in vitro, Dox@HFn Gel was placed in a 5 mL EP tube. 1 mL of PBS buffer solution with pH 7.4, 6.8, and 5.0, respectively, was added to each EP tube. The tubes were then cultured in a shaker bath at 37 °C. At each setting time point, 500 µL of the above solution was taken out for fluorescence detections, and 500 µL of buffer solution was immediately added into original solution. The cumulative release of Dox in the gel at different time points was measured by UV–vis absorption.

### In Vivo Evaluation of Hydrogel Degradation

First, hydrogel solution (50 µL) was injected into the back of BALB/c mice. The mice were then euthanized at setting time points (1 h and 7, 14, and 21 days after treatment). The remaining hydrogel in each mouse was photographed and the weight was recorded.

### Inhibition of Cellular Uptake by Endocytosis Inhibitors

4T1 cells (1×10^5^ cells/well) were seeded into a 24‐well plate in complete DMEM medium for 12 h. The old medium was then removed, the 4T1 cells were pretreated with 500 µL of serum‐free DMEM medium in per well for 1 h, which contained Amirolide (200 µm) inhibiting micropinocytosis‐mediated endocytosis, Chlorpromazine (20 µm) inhibiting a clathrin‐mediated endocytosis, and Genistein (700 µm) inhibiting caveolae‐mediated endocytosis, respectively. After the pretreatment, Dox@HFn (5 µm) was added to each well, and cells were incubated for 4 h. 4T1 cells were then digested with 0.25% trypsin at 37 °C for 2 min in per well. 4T1 cells were collected in an EP tube and centrifuged at 3000 g for 3 min. Following the removal of the supernatant, the 4T1 cells were resuspended in a 200 µL PBS solution for flow cytometry detection (BD Biosciences, USA).

### Formation of Multicellular Tumor Spheroids

The 4T1 cell‐derived multicellular spheroids were cultured using agarose gel‐bottomed six‐well plate. When the diameter of cell spheroids reached the range of 100–150 µm, cell spheroids were planted in agarose gel‐bottomed 24‐well plate and treated with 10 µm Dox or Dox@HFn for 4 h. To understand the role of ferritin in penetration, cell spheroids were pretreated with ten times HFn for 2 h to block cell surface ferritin receptors. When the treating time was up, cell spheroids were washed twice with PBS and observed using CLSM (Ti‐E A1, Nikon, Japan). For quantitative analysis, after cell spheroids were treated for 4 h, the cell spheres were washed three times with PBS and digested with 0.25% trypsin. The cells were collected in EP tube and centrifuged at 3000 g for 3 min. The supernatant was discarded, and the cells were suspended with 200 µL PBS for flow cytometry detection (BD Biosciences, USA).

### Detection of CALR Exposure

4T1 cells were seeded in 24‐well plates with a density of 1 × 10^5^ cells/well for 12 h. The cells were then treated with PBS, HFn, Dox (2 µm) or Dox@HFn (10 µm) for 24 h. After digestion and collection, the cells were blocked with CD16/32 antibody (Biolegend101320) for 15 min and then incubated with anti‐CALR‐AF647 antibody (ab196159) at 4 °C for 30 min. Then the cells were washed twice and the expression calreticulin of cell on the surface was detected by flow cytometry (BD Biosciences, USA).

### Detection of Extracellular ATP

4T1 cells were seeded in a 24‐well plate at a density of 1×10^5^ cells/well for 12 h. Then, the cells were treated with PBS, HFn, Dox (2 µm) or Dox@HFn (10 µm) for 24 h. The cell culture supernatant was collected and centrifuged at 12 000 g for 5 min at 4 °C. Next, the cell culture supernatant was mixed with an ATP detection working solution (Beyotime, #S0026). The chemiluminescence was detected by enzyme plate analyzer. The ATP concentration can be calculated from the standard curve.

### Detection of Extracellular HMGB1

4T1 cells were seeded into a 24‐well plate at a density of 1×10^5^ cells per for 12 h. After that, the cells were treated with PBS, HFn, Dox (2 µm), or Dox@HFn (10 µm) for 24 h. The cell culture supernatant was collected and centrifuged at 12 000 g for 5 min at 4 °C. The amount of HMGB1 secreted by the cells was quantitatively detected using an HMGB1 ELISA kit (Arigo, #81351).

### Transcytosis of Dox@HFn

The classic transwell detection system was used. 4T1 cells were planted in 24‐well plates at a density of 1×10^5^ cells per well overnight. Then, 4T1 cells were treated with Dox@HFn (10 µm) with or without exocytosis inhibitor EXO1 for 4 h. The cells were washed for three times with PBS, then digested and added into the upper chamber. After 4 h, the cells in lower chamber were washed for three times with PBS and fixed for 15 min with 4% paraformaldehyde. After washing for three times with PBS, the cells were in per well added 0.2 mL 1×DAPI for 10 min to stain the cell nuclei. Cell climbing tablets were imaged by CLSM (Ti‐E A1, Nikon, Japan). For quantitative analysis, the cells in lower chamber were washed three times with PBS and digested with 0.25% trypsin for ≈5 min. The cells were collected in EP tube, centrifuged at 3000 g for 3 min, and resuspended with PBS for flow cytometric analysis (BD Biosciences, USA)

### Bone Marrow‐Derived Dendritic Cells (BMDCs) Extraction

BALB/c mice (female, 6 weeks old) were euthanized. The femur and tibia were carefully separated from mice under aseptic conditions, and the bone marrow cavity was rinsed with PBS. The bone marrow cells were filtered through a 200‐mesh filter and centrifuged at 450 g for 5 min. Red blood cells in bone marrow cells were removed using the red blood cell lysis buffer (Beyotime, C3072). The bone marrow cells were resuspended in complete RPMI1640 medium with containing mouse IL‐4 (10 ng mL^−1^) and mouse GM‐CSF (10 ng mL^−1^). The cell concentration was adjusted to 5×10^5^ cells mL^−1^. The bone marrow cells were seeded in a 24‐well plate at a density of 5×10^6^ cells/well for 7 days.

### In Vitro BMDC Activation Experiments

First, 4T1 cells were treated with PBS, HFn, Dox (2 µm) or Dox@HFn (10 µm) for 24 h. Then, 4T1 cells were co‐cultured with BMDC for 24 h. The harvested BMDCs were blocked with anti‐CD16/32 antibody (diluted at 1:100) for 15 min and then labeled with fluorescent‐conjugated antibodies (anti‐CD11c (Biolegend, 506904), anti‐CD86 (Biolegend, 105012), anti‐CD80 (Biolegend, 107406), and anti‐MHC‐II (Biolegend, 107632)) for 30 min. After washing with PBS, the cells were resuspended in PBS for flow cytometric analysis (BD Biosciences, USA).

### T Cell Activation Assay

4T1 cells were treated with PBS, HFn, Dox (2 µm) or Dox@HFn (10 µm) for 24 h. Then, 4T1 cells were co‐cultured with BMDC for 24 h. Subsequently, T cells and BMDC were cocultured at a 10:1 ratio for an additional 24 h. CD69 expression was detected by flow cytometry (BD Biosciences, USA). The supernatants obtained from the co‐culturing of T cells and DC for 24 h were used for cytokine analysis. The levels of IFN‐γ were measured using the Dakewe biotech Mouse IFN‐γ ELISA kit, following the manufacturer's instructions. Similarly, the levels of TNF‐α were determined using the Dakewe biotech Mouse TNF‐α ELISA kit, following the manufacturer's guidelines.

### In Vivo Antitumor Studies

To establish an orthotopic breast cancer model, female BALB/c mice (6 weeks old) were injected with 4T1 cells (5 × 10^5^) directly into their mammary fat pads. The effects of Dox@HFn Gel were then evaluated in this model. Once the tumor volume reached ≈50 mm^3^, the tumor‐bearing mice were randomly divided into four groups and treated with PBS, Dox (5 mg kg^−1^), and Dox@HFn Gel (Dox: 5 mg kg^−1^). The tumor growth of mice was measured every two days. The tumor volume was calculated with V = a×b^2^/2 (a represents the long diameter, and b represents the minor axis).

### TUNEL Staining

Section paraffin blocks of tumor tissues were sectioned to 4 µm thickness. The sections were transferred to the slides. The slides were baked at 60 °C after dewaxing with xylene, hydrated with gradient ethanol and washed with deionized water for 2 min. 30 µg mL^−1^ DNase‐free protease K was added dropwise in the slides at 37 °C for 15 min. The slides were washed three times with PBS. 30 µL of TUNEL assay solution was added to the slides at 37 °C for 90 min under dark. The slides were washed with PBS, and DAPI stained the cell nuclei for 10 min. The slides were washed three times with PBS again and imaged by CLSM (Ti‐E A1, Nikon, Japan).

### Immune Cells Analysis after the Treatment

4T1 mouse breast cancer model was constructed. Tumor‐bearing mice were treated with PBS, Dox‐aPD‐1 (5 mg kg^−1^ Dox, single injection), Dox@HFn Gel (5 mg kg^−1^ Dox), and Combo (5 mg kg^−1^ Dox), respectively. aPD‐1 (100 µg per mice) was intraperitoneal administered on days 1, 3, and 5. Lymph nodes were harvested in tumor‐bearing mice on the 9th day after various treatments. The lymph nodes were ground into single cells. The cells in lymph node were blocked with CD16/32 antibodies and then labeled with fluorescent‐conjugated antibodies (α‐CD11c (Biolegend, 506904), α‐IA/IE (Biolegend, 107622), α‐CD80 (Biolegend, 107406), α‐CD86 (Biolegend, 105012), α‐CD45.2 (Biolegend, 109838) and α‐CD11b (Biolegend, 101259)) for 30 min. The cells in lymph node were detected by flow cytometry (BD Biosciences, USA).

### Immunofluorescence Analysis

Tumor tissues were collected on the 9th day of treatment. Tumor tissues were fixed with 4% paraformaldehyde for 4 h, then tumors were transferred to a 30% sucrose solution and dehydrated at 4 °C overnight. Tumor tissues were embedded with OCT glue. Tumor tissues were then sectioned to a thickness of 4.5 µm. After blocking with PBS containing 10% serum for 1 h at room temperature, the tumor tissue was stained with primary antibody (anti‐mouse CD8 (Abcam, ab217344), anti‐HMGB1 (Abcam, ab79823) and anti‐calreticulin (Abcam, ab92516)) overnight. Tumor tissue was treated with secondary antibodies (Abcam, ab175695) for 2 h, stained with DAPI for 10 min, and imaged by CLSM (Ti‐E A1, Nikon, Japan).

### Cytokine Detection

Tumor tissues were harvested in tumor‐bearing mice on the 9th day of various treatment and ground into a single‐cell suspension which was centrifuged to collect the cell supernatant. The cell supernatant was used to detect cytokine concentrations. The levels of intratumor IFN‐γ (Dakewe biotech), IL‐6 (Dakewe biotech), and TNF‐α (Dakewe biotech) were detected by ELISA kits.

### Analysis of Long‐Term Immune Memory

To establish an orthotopic breast cancer model, female BALB/c mice (6 weeks old) were injected with 4T1 cells (5 × 10^5^) directly into their mammary fat pads. The spleen was collected on the 40th day after various treatment and ground into single cells. Red blood cells in splenocytes were removed with the red blood cell (RBC) lysis buffer. Splenocytes were blocked by CD16/32 antibodies and then labeled with fluorescent‐conjugated antibodies (anti‐CD45 (Biolegend, 103134), anti‐CD44 (Biolegend, 103018), (anti‐CD62L (Biolegend, 104428), anti‐CD8 (Biolegend, 100742) and anti‐CD3 (Biolegend, 100306)) for 30 min. Following washing with PBS buffer, splenocytes were resuspended with PBS for flow cytometric analysis.

### H&E Staining

Section paraffin blocks of tumor tissues were sectioned to 4 µm thickness. The sections were transferred to the slides. The slides were baked at 60 °C after dewaxing with xylene, hydrated with gradient ethanol, and washed with deionized water for 2 min. First, hematoxylin was used to stain the cell nuclei for 1 min. Then the slides were equalized with 80% ethanol for 2 min, stained cytoplasm with eosin for 20 s, and bleached with 80% ethanol for 2 min. After ethanol dehydration with a gradient ascending concentration, the slides were imaged by digital pathology microscope slide scanners.

### Tumor Resection and Metastasis in 4T1 Model

To establish an orthotopic breast cancer model, female BALB/c mice (6 weeks old) were injected with 4T1 cells (5 × 10^5^) directly into their mammary fat pads. When the tumor volume in mice reached ≈300 mm^3^, the surgical area was disinfected with 75% alcohol, and 90% of the tumor was removed surgically. Then, surgical mice were randomly divided into four groups and treated with PBS, Dox‐aPD‐1, Dox@HFn Gel^L^, and Combo, respectively. Tumor recurrence and weight of mice were monitored every two days after various treatment. Furthermore, the lungs of the mice in each group were collected and the number of pulmonary metastatic nodules were counted by visual observation on the 40th day after various treatment.

### Glioblastoma Resection Model

To establish an orthotopic GL261 tumor‐bearing mouse model, luciferase‐expressing GL261 cells (2 × 10^5^ cells per mouse in 3 µL of PBS) were injected into the brains (2 mm right, 1 mm anterior to the bregma, 2 mm depth) of C57BL/6J mice. The GBM was surgically removed on day 8 after inoculation. Subsequently, each mouse received an injection of 10 µL of Dox@HFn Gel into the tumor resection cavity on day 8. Intraperitoneal administration of αCD47 (50 µg per mouse) was performed on days 9, 11, and 13. After intravenous injection of D‐fluorescein (150 mg kg^−1^) into mice, bioluminescence imaging of GMB was performed using an imaging system (PerkinElmer IVIS, America). Mice survival after the treatment was also recorded.

### Statistical Analysis

GraphPad Prism 8.0 software was applied for statistical analysis. Statistical analysis was analyzed using an unpaired Student's *t*‐test (two‐tailed) for two groups, and one‐way ANOVA with Dunnett's multiple comparisons. The statistical significance was present by asterisks, *P < 0.05, **P < 0.01, ***P < 0.001, ^****^ *P* < 0.0001.

## Conflict of Interest

The authors declare no conflict of interest.

## Author Contributions

R.L. and Q.L. contributed equally to this work. J.D. and K.F. conceived and supervised the project. R.L., Q.L., J.L., Y.L., and X.Z. performed the experiments. R.L., K.F., and J.D. wrote the paper. All authors discussed the data and reviewed the manuscript.

## Supporting information

Supporting Information

## Data Availability

The data that support the findings of this study are available from the corresponding author upon reasonable request.
