# Supplementary material for: Ferritin‐Based Nanocomposite Hydrogel Promotes Tumor Penetration and Enhances Cancer Chemoimmunotherapy
Source: Adv Sci (Weinh). 2023 Nov 29;11(3):2305217. doi: 10.1002/advs.202305217 (PMC10797422; doi:10.1002/advs.202305217)
Supplement: Supplementary file 1 — Supporting Information [file ADVS-11-2305217-s001.pdf]

ADVANCED SCIENCE
Open Access

Supporting Information

for *Adv. Sci.*, DOI 10.1002/advs.202305217

Ferritin-Based Nanocomposite Hydrogel Promotes Tumor Penetration and Enhances Cancer Chemoimmunotherapy

Rong Liu, Qian Liang, Jia-Qi Luo, Yu-Xuan Li, Xin Zhang, Kelong Fan and Jin-Zhi Du**

Supporting Information for

Ferritin-based Nanocomposite Hydrogel Promotes Tumor Penetration and Enhances Cancer Chemoimmunotherapy

Rong Liu[#], Qian Liang[#], Jia-Qi Luo, Yu-Xuan Li, Xin Zhang, Kelong Fan[*], and Jin-Zhi Du[*]

R. Liu, J. Luo, X. Zhang, J. Du

School of Medicine, South China University of Technology, Guangzhou 510006, China

Email: djzhi@scut.edu.cn

Q. Liang, K. Fan

CAS Engineering Laboratory for Nanozyme, National Laboratory of Biomacromolecules, Institute of Biophysics, Chinese Academy of Sciences, Beijing 100101, China

Email: fankelong@ibp.ac.cn.

Y. Li

School of Biomedical Sciences and Engineering, Guangzhou International Campus, South China University of Technology, Guangzhou 511442, China

K. Fan

University of Chinese Academy of Sciences, Beijing 101408, China.

Nanozyme Medical Center, School of Basic Medical Sciences, Zhengzhou University, Zhengzhou 450052, China.

J. Du

National Engineering Research Center for Tissue Restoration and Reconstruction, South China University of Technology, Guangzhou 510006, China.

Key Laboratory of Biomedical Materials and Engineering of the Ministry of Education, and Guangdong Provincial Key Laboratory of Biomedical Engineering, South China University of Technology, Guangzhou 510006, China.

[#]These two authors contributed equally to this work.

Experimental Section

Preparation and characterization of HFn and DOX@HFn: HFn and doxorubicin (Dox) loaded HFn were synthesized as previously described.[1] The average hydrodynamic diameter sizes of the HFn and DOX@HFn were determined by dynamic light scattering (DLS).

Oxidation of dextran (Dex-CHO): First, 500 mg of dextran was added into moderate amount of deionized water to prepare a 10% (w/v) aqueous solution. Next, 4 mL of $NaIO_4$ (0.5 M) solution was added into above prepared aqueous solution. After that, the mixture was stirred for 4 h at room temperature in dark condition. Sequentially, 1 mL of ethylene glycol was added and stirring for 2 h to terminate the reaction. Then, above solution was transferred to 7000 Da dialysis bags and dialyzed with deionized water for 5 days. Finally, the solution was lyophilized to obtain a white flocculent solid product with a yield of 95%.

Determination of oxidation of dextran: Dex-CHO powder (30.9 mg) was added into 8 mL of hydroxylamine hydrochloride solution (0.25 N, containing 0.007% methyl orange). The resulting solution was stirred at room temperature for 2 h. Afterward, this solution was titrated with standard solution of 0.1 N NaOH. The pH values of above solution and dosage of NaOH were recorded for plotting the curves.

Determination of hydrogel swelling ratio: First, the hydrogel was freeze-dried and weighed. Then, it was soaked in PBS for a specific period of time. After removal from the PBS, the excess surface water was gently blotted using absorbent paper, and the hydrogel was reweighed. The swelling mass of the hydrogel at different time points is

Wt (it is 1, 2, 4...), the mass of the hydrogel before PBS soaking is W_0. The swelling ratio of the hydrogel is $(Wt - W_0)/ W_0 \times 100\%$.

In vitro uptake of Dox@HFn: Flow cytometry detection: 4T1 cells were seeded plate with a density of 1×10^5 cells/well overnight. The next day, 4T1 cells in each well were treated with 5 μM Dox or Dox@HFn in serum-free medium at 37 °C for 1, 2 and 4 h, respectively. When the treating time was up, the cells were collected by washing twice with PBS and then digested with 0.25% trypsin. Following an additional two washes with PBS, the fluorescence intensity of cells in the PE channel was detected by flow cytometry (BD Biosciences, USA), which represented the intracellular Dox content.

Confocal fluorescence microscopy: Glass coverslips were placed in a 24-well plate, and 4T1 cells were seeded with a density of 5×10^4 cells/well. The following day, the cells in each well were treated with either 5 μM Dox or Dox@HFn in serum-free medium for 4 h at 37 °C. When the treating time was up, the cells were washed for twice with PBS and were fixed with 4% paraformaldehyde for 10 min at room temperature. Four percent paraformaldehyde solution was then discarded and the glass coverslips were washed twice with PBS buffer. The cell nuclei were stained with DAPI. After two washes with PBS buffer, glass coverslips were imaged by CLSM (Ti-E A1, Nikon, Japan).

MTT assay: 4T1 cells were seeded in a 96-well plate with a density of 5×10^3 cells/well overnight. 4T1 cells were then treated with Dox or Dox@HFn at a concentration gradient of 20, 10, 5, 2.5, 1.25, 0.625, 0.312, 0.156, 0.078 and 0 μM for

48 h. 10 μL of MTT solution (5 mg/mL, Beyotime, #ST316) was added to per well. The plate was then incubated for 4 h in the dark. The cell supernatant was carefully aspirated, and 100 μL of DMSO was added to each well. The plate was shaken in the dark for 10 minutes to dissolve the formazan crystals. The absorbance of the solution in the plate was measured at 560 nm using a microplate reader (BioTek Instruments, Winooski, VT, USA).

Detection of cell apoptosis: 4T1 cells were plated with 24-well plates with a density of 1×10^5 cells/well overnight. 4T1 cells were treated with PBS, HFn, Dox (2, 5 μM) or Dox@HFn (5, 10 and 15 μM) for 48 h. After the incubation period, the cells were harvested and stained with Annexin V-APC and DAPI (Multi Sciences, #AP105-100) for flow cytometry analysis (BD Biosciences, USA).

In vitro phagocytosis of BMDMs: BMDMs separated from BALB/c mice were stained with eFluor 670 and seeded in 24-well plates. 4T1 cells were treated with PBS, HFn, Dox (2 μM) or Dox@HFn (10 μM) for 24 h. Tumor cells labeled with CFSE were cocultured with eFluor 670-labeled BMDMs for 12 h. All cells were then collected, washed, and analyzed by flow cytometry. Phagocytosis percentage was determined by the percentage of dual positive BMDMs (CFSE eFluor 670) within the total BMDMs population (eFluor 670).

Reference:

[1] K. Fan, X. Jia, M. Zhou, K. Wang, J. Conde, J. He, J. Tian, X. Yan, *ACS Nano* **2018**, 12, 4105-4115.

Supplemental Data

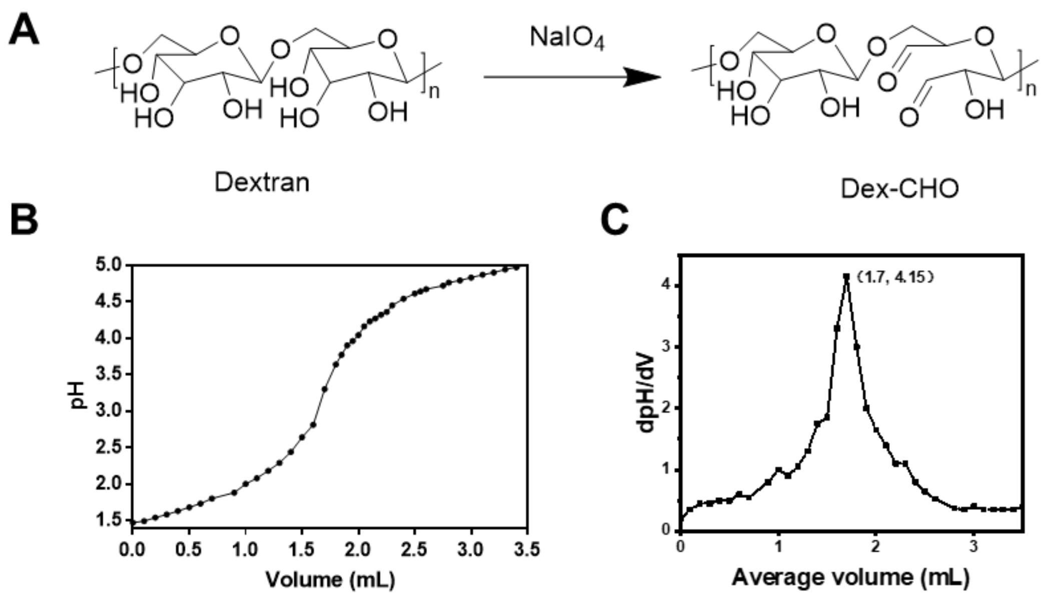

Figure S1. A) Synthetic procedure of Dex-CHO. B) and C) Titration differential curve for the determination of Dex-CHO oxidation degree by hydroxylamine hydrochloride potentiometric titration.

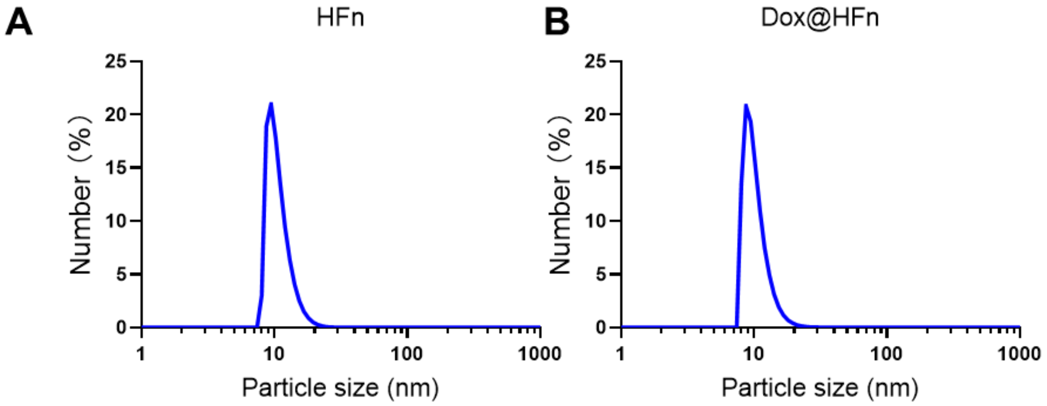

Figure S2. A) Particle size distribution of HFn and B) Dox@HFn.

Table S1. The gelation time of the HFn Gel.

Sample	HFn (mg/mL)	Dex-CHO (mg/mL)	Volume ratio (v/v)	Gelation time (min)
1	200	150	1:1	2
2	200	130	1:1	3
3	200	120	1:1	5
4	200	100	1:1	7
5	200	90	1:1	11

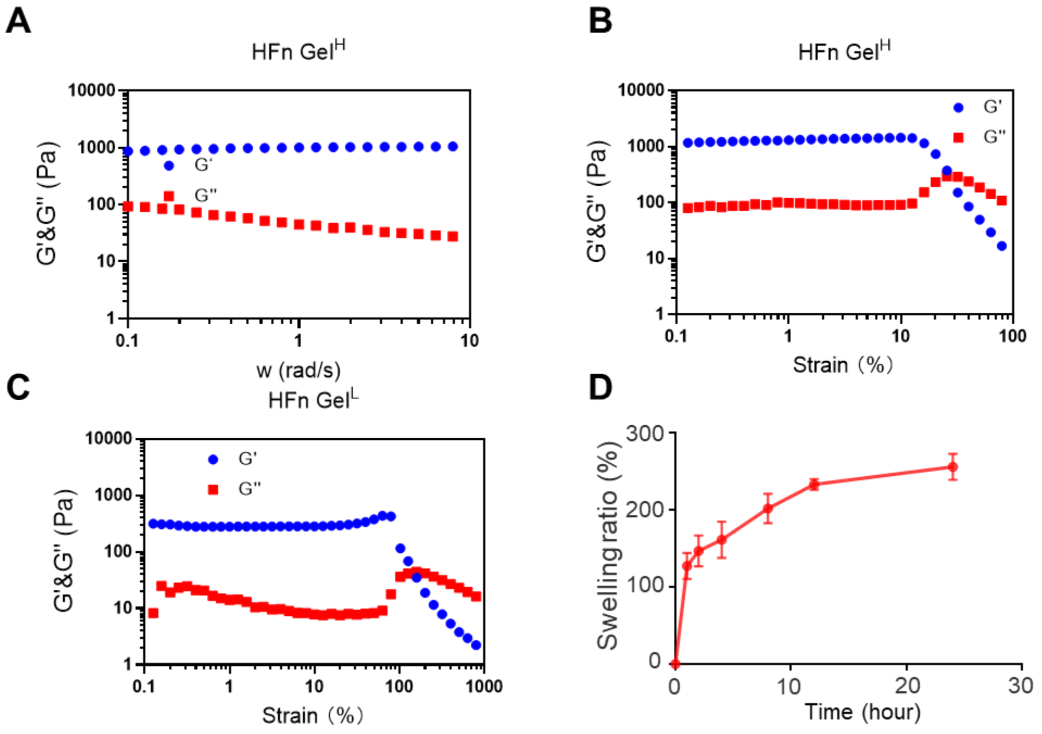

Figure S3. A) The spectrum of the storage modulus (G') and loss (G'') modulus of HFn Gel^{H} with frequency. B) The spectrum of the storage modulus (G') and loss (G'') modulus of HFn Gel^{H} and C) HFn Gel^{L} with strain. D) The swelling ratio of Dox@HFn Gel^{L} as a function of time in PBS buffer (n = 3).

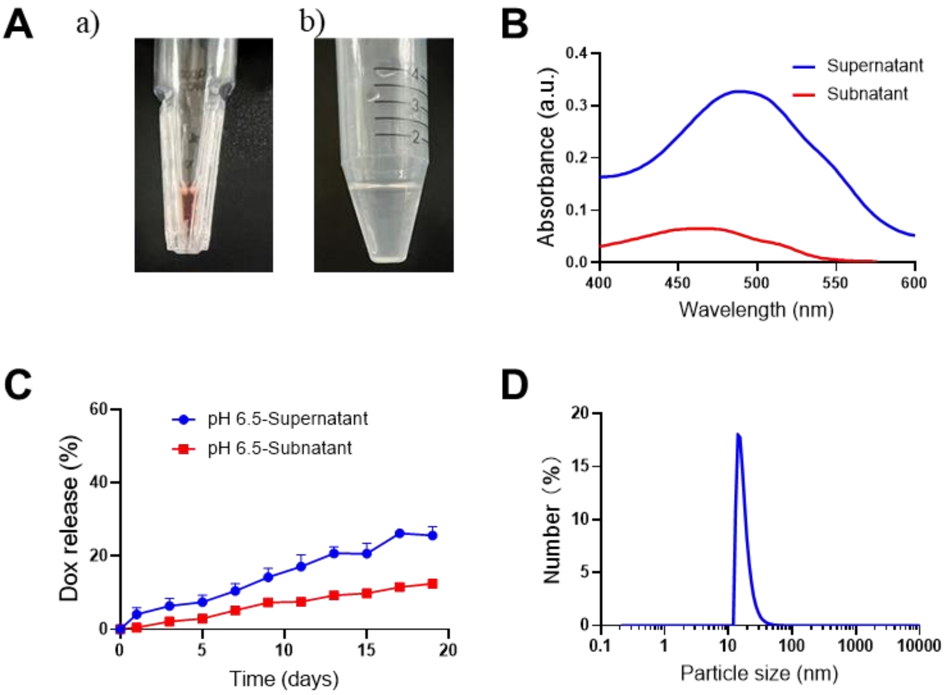

Figure S4. A) The solution obtained after ultrafiltration of the external media for drug release. a) the supernatant and b) the subnatant after ultrafiltration. B) UV-visible absorption plot of the supernatant and subnatant after ultrafiltration. C) *In vitro* the accumulative release of Dox from the Dox@HFn Gel in PBS at pH 7.4 in the supernatant and subnatant after ultrafiltration. D) Particle size distribution of Dox@HFn in the supernatant.

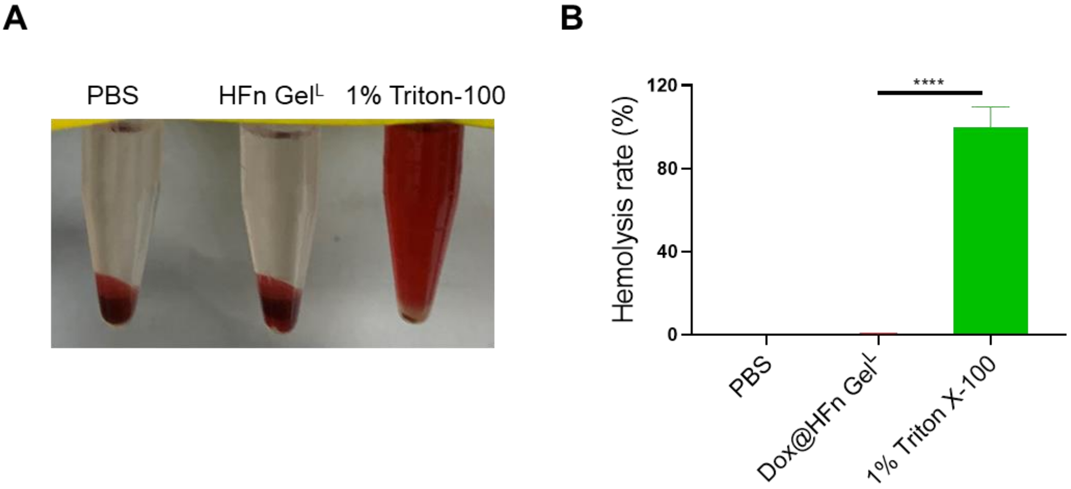

Figure S5. The hemolysis of HFn Gel^{L}. A) Photograph of supernatant after centrifugation of each group and B) hemolysis rate of the hydrogel (n = 3).

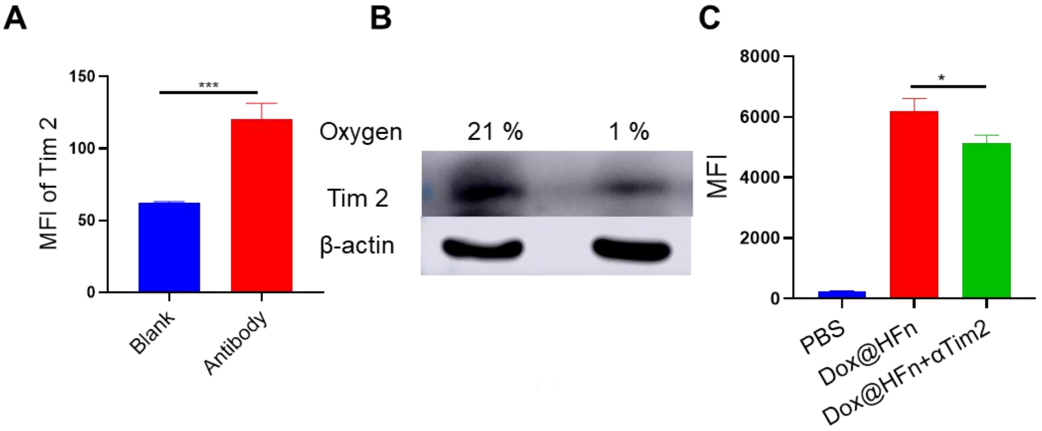

Figure S6. A) The expression of Tim-2 receptor on the surface of 4T1 cells was detected by flow cytometry (n = 3). B) Western blot analysis was performed to detect Tim-2 expression in 4T1 cells that were incubated under normoxic conditions (21% O_2) or hypoxic conditions (1% O_2). C) Tim-2 dependent binding of Dox@HFn to 4T1 cells in the presence of Tim-2 antibody by flow cytometry (n = 3).

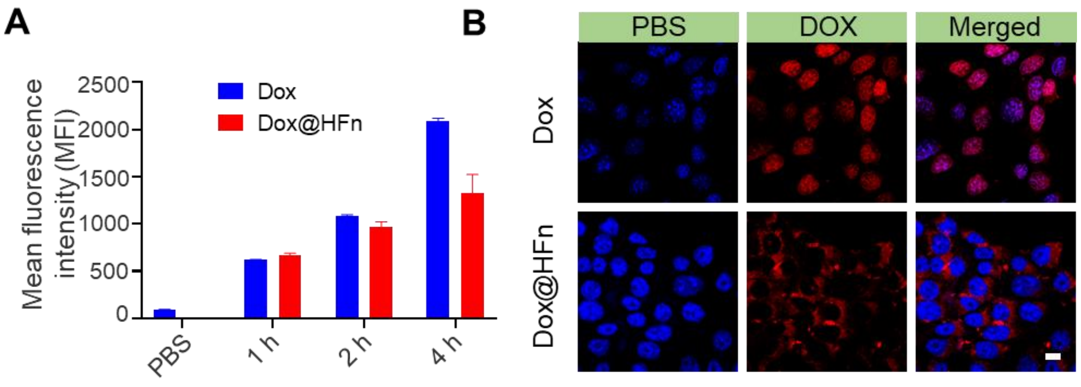

Figure S7. A) Flow cytometry analysis of cellular uptake for free Dox or Dox@HFn by 4T1 cells at different periods (n = 3). B) CLSM images of cellular uptake of free Dox or Dox@HFn by 4T1 cells at 4 h after treatment. Scale bars: 10 μm.

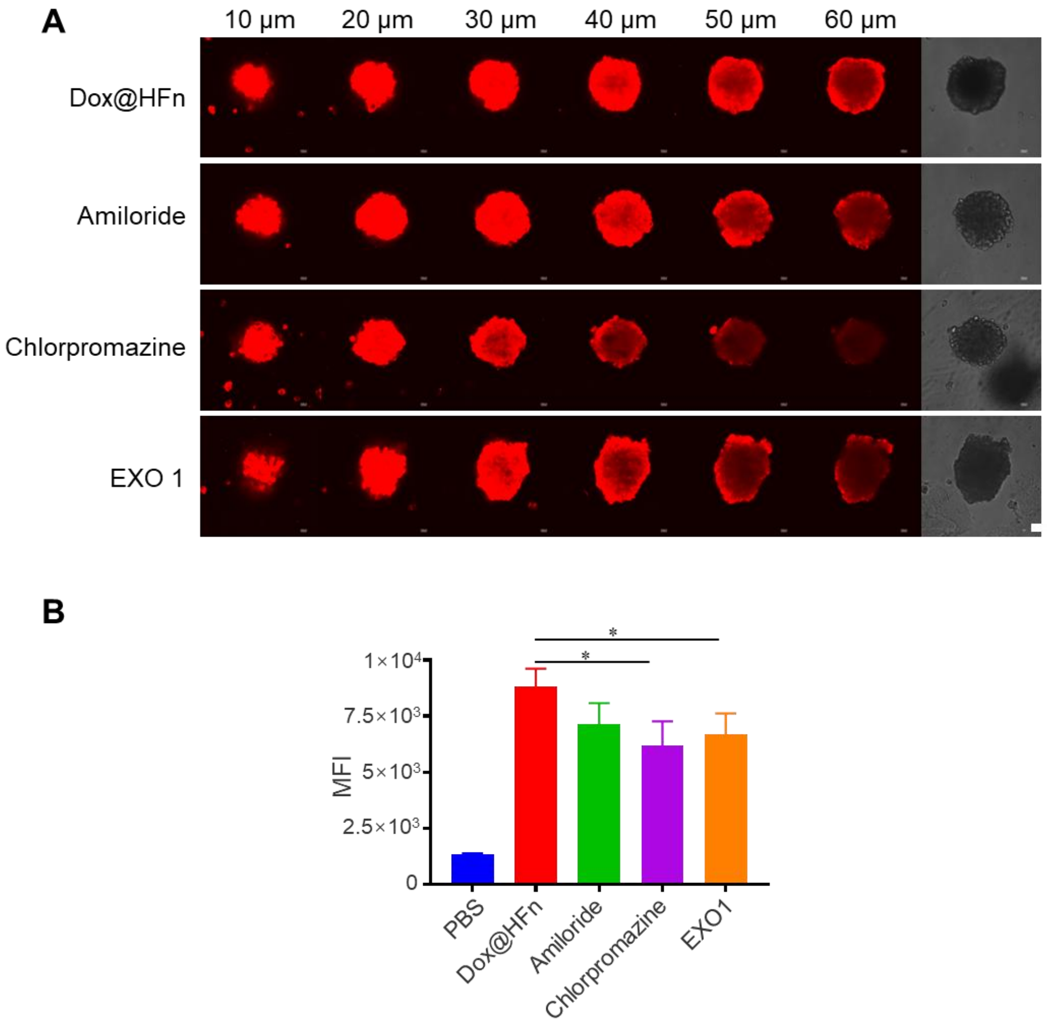

Figure S8. A) CLSM images and B) flow cytometry analysis of the 4T1 cell-derived multicellular spheroids with various treatments for 4 h (n = 3). Scale bars: 50 μm. Statistical data are presented as means ± SD. *$P < 0.05$.

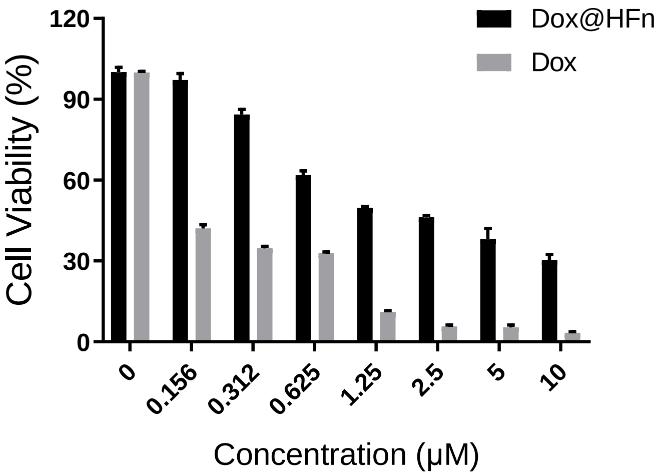

Figure S9. Cell viability of 4T1 cells incubated with free Dox and Dox@HFn at various Dox concentrations for 48 h (n = 3).

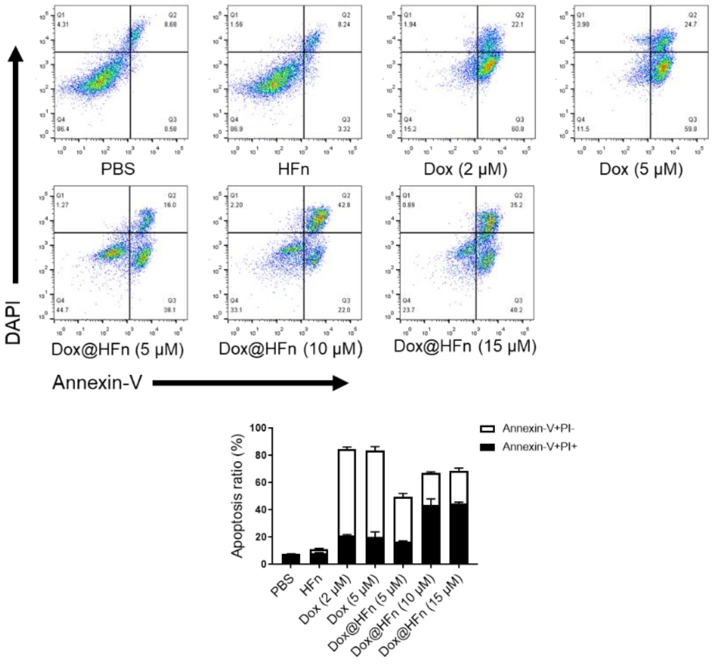

Figure S10. 4T1 cells were co-incubated with free Dox or Dox@HFn for 48 h, and apoptosis was detected using Annexin V/DAPI double staining by flow cytometry (n = 3).

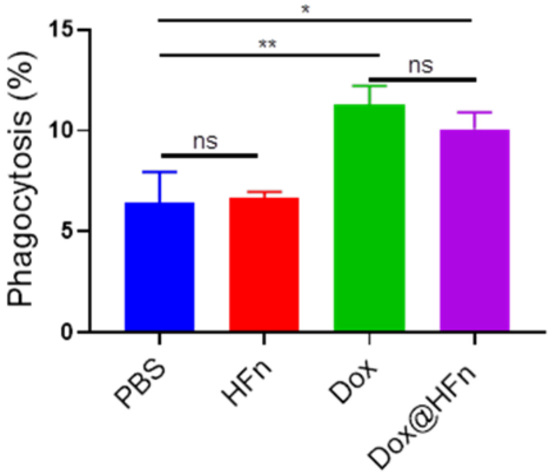

Figure S11. The phagocytosis rates of various treatments treated 4T1 cells by BMDM (n = 3). The BMDM were cocultured with 4T1 cells for 24 h with different treatments.

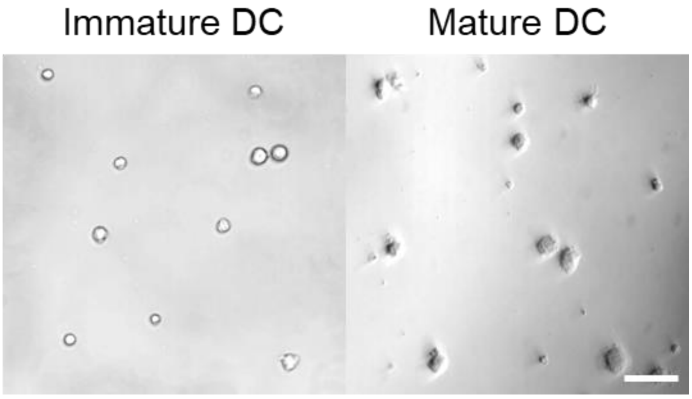

Figure S12. Morphological images of BMDCs before or after co-culturing with Dox@HFn-treated 4T1 cells for 24 h. Scale bars: 50 μm.

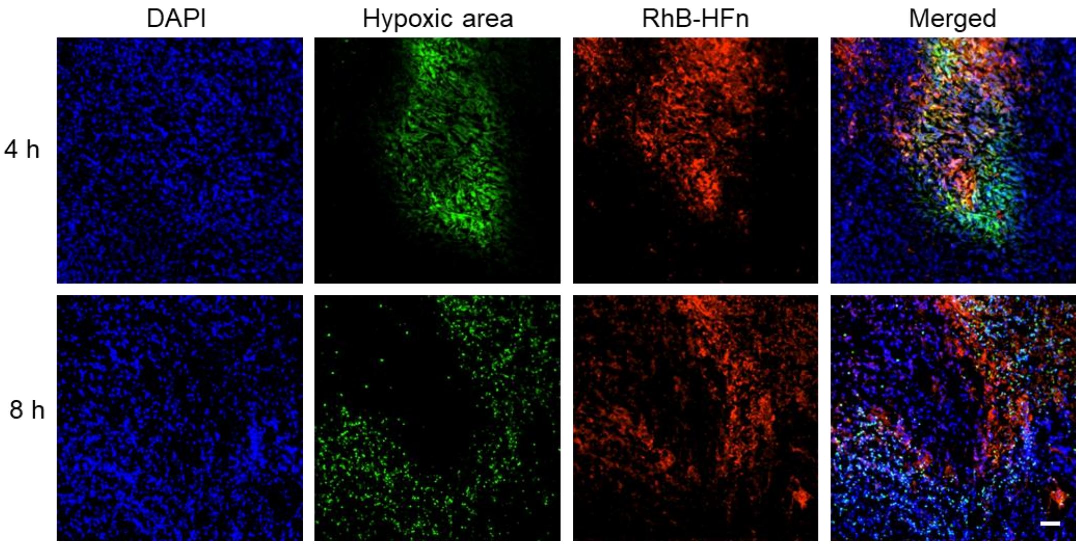

Figure S13. Representative CLSM images showing time-course tumor accumulation of systemically administered HFn (red) in hypoxic tumor areas (green). Blue colors represent cell nuclei. Scale bars: 50 μm.

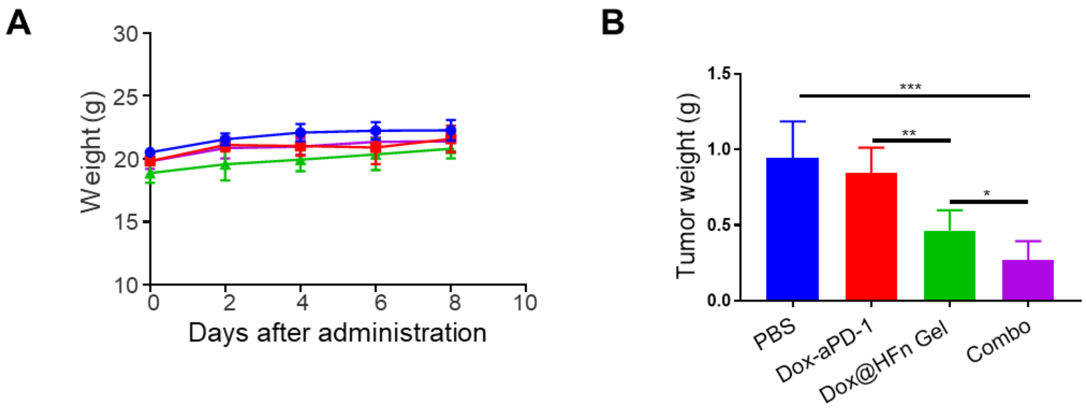

Figure S14. A) Weight of 4T1 tumor-bearing mice from various groups of mice (n = 5). B) Weights of 4T1 tumor harvested from various groups of mice on the 9th day (n = 5).

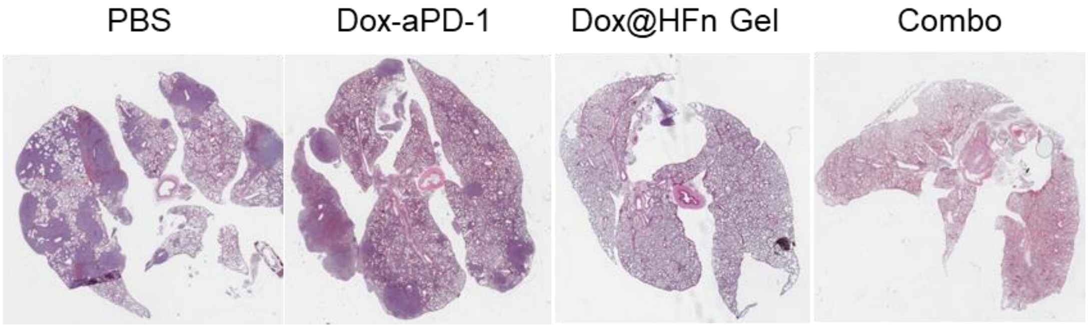

Figure S15. H&E staining of pulmonary tissue was performed on the 40th day after tumor cell inoculation.
